# Genome-Wide Characterization of Serine/Arginine-Rich Gene Family and Its Genetic Effects on Agronomic Traits of *Brassica napus*

**DOI:** 10.3389/fpls.2022.829668

**Published:** 2022-02-16

**Authors:** Meili Xie, Rong Zuo, Zetao Bai, Lingli Yang, Chuanji Zhao, Feng Gao, Xiaohui Cheng, Junyan Huang, Yueying Liu, Yang Li, Chaobo Tong, Shengyi Liu

**Affiliations:** The Key Laboratory of Biology and Genetic Improvement of Oil Crops, The Ministry of Agriculture and Rural Affairs of the PRC, Oil Crops Research Institute of the Chinese Academy of Agricultural Sciences, Wuhan, China

**Keywords:** serine/arginine-rich gene family, *Brassica napus*, expression pattern, alternative splicing, association mapping analysis, agronomic traits

## Abstract

Serine/arginine-rich (SR) proteins are indispensable factors for RNA splicing, and they play important roles in development and abiotic stress responses. However, little information on *SR* genes in *Brassica napus* is available. In this study, 59 *SR* genes were identified and classified into seven subfamilies: SR, SCL, RS2Z, RSZ, RS, SR45, and SC. In each subfamily, the genes showed relatively conserved structures and motifs, but displayed distinct expression patterns in different tissues and under abiotic stress, which might be caused by the varied *cis*-acting regulatory elements among them. Transcriptome datasets from Pacbio/Illumina platforms showed that alternative splicing of *SR* genes was widespread in *B. napus* and the majority of paralogous gene pairs displayed different splicing patterns. Protein-protein interaction analysis indicated that SR proteins were involved in the regulation of the whole lifecycle of mRNA, from synthesis to decay. Moreover, the association mapping analysis suggested that 12 *SR* genes were candidate genes for regulating specific agronomic traits, which indicated that *SR* genes could affect the development and hence influence the important agronomic traits of *B. napus*. In summary, this study provided elaborate information on *SR* genes in *B. napus*, which will aid further functional studies and genetic improvement of agronomic traits in *B. napus*.

## Introduction

RNA splicing is an important process in eukaryotes that could produce one or multiple mature mRNAs via different splicing sites, which significantly increases the flexibility of gene expression regulation and the diversity of transcriptome and proteome (Black, 2003). The process is mediated by the spliceosome, a large macromolecule complex composed of five small nuclear ribonucleo-proteins (snRNPs) and a mass of proteins (Will and Luhrmann, 2010). Among these proteins, the serine/arginine-rich (SR) proteins are vital splicing factors to regulate the selections of splicing sites by binding splicing enhancers on the pre-mRNA (Zahler et al., 1992). The structure of SR proteins is conserved, containing one or two RNA binding domains (RBDs) at the N-terminus and an arginine/serine-rich (RS) domain at the C-terminus (Shepard and Hertel, 2009). The RBDs are responsible to recognize and bind to specific RNA regions, while the RS domain contributes to the protein-protein interactions. The subcellular localization of SR proteins is directly related to their molecular functions, and it has been reported that they are localized in the nuclear speckles (Caceres et al., 1997), a subset of them could shuttle between the nucleus and cytoplasm (Sapra et al., 2009).

In plants, SR proteins were initially identified in Arabidopsis (Kalyna and Barta, 2004), then in rice, maize, wheat, tomato, cassava, and so on (Isshiki et al., 2006; Richardson et al., 2011; Yoon et al., 2018; Chen et al., 2019, 2020b; Gu et al., 2020; Rosenkranz et al., 2021). According to sequence similarity, SR proteins could be divided into seven subfamilies: SR, SCL, RS2Z, RSZ, RS, SR45, SC, and three of them (SCL, RS2Z, RS) are plant-specific (Richardson et al., 2011). Subfamily SCL is the largest plant-specific one containing members from dicots, monocots, moss, and green algae. Subfamily RS2Z was mainly composed of dicots and monocots, whereas most members of subfamily RS came from photosynthetic eukaryotes. Many studies have shown that the *SR* genes play important roles in plant developmental processes and respond to hormonal signaling or environmental stress (Isshiki et al., 2006; Palusa et al., 2007; Reddy and Shad Ali, 2011; Melo et al., 2020). For example, the life cycle of *Atsr45-1* was significantly shorter, the leaves of *Atsr45-1* were elongated and curly, and the number of petals and stamens was also significantly different from the wild type (Ali et al., 2007). Overexpression of *RSZ33* in Arabidopsis can result in developmental abnormalities in embryos and root apical meristem (Kalyna et al., 2003). And knockout SC and SCL in Arabidopsis could affect the transcriptions of many genes, resulting in serrated leaves, late flowering, shorter roots and abnormal silique phyllotaxy (Yan et al., 2017). Most members of the plant-specific SCL are involved in stress responses mediated by exogenous abscisic acid (ABA) (Cruz et al., 2014). In terms of environmental stress, *Atsr34B* reduces plant tolerance to calcium by regulating the expression of *IRT1* (Zhang et al., 2014), while *AtRS40* and *AtRS41* act as critical modulators under salt stress (Chen et al., 2013).

In addition to regulating the splicing of other genes, *SR* genes also could be alternatively spliced. A total of 19 *SR* genes were identified in Arabidopsis (Kalyna and Barta, 2004). Among them, 15 genes could produce 95 transcripts under hormone induction or abiotic stress, which greatly increased the complexity of the *SR* genes by sixfold (Palusa et al., 2007). There were 21 and 18 *SR* genes in maize and sorghum, respectively, whereas 92 and 62 transcripts were detected in each of them, and the majority of SR transcripts were not conserved between maize and sorghum (Rauch et al., 2014). *SR* genes in tomato showed different splicing profiles in various organs as well as in response to heat stress (Rosenkranz et al., 2021). And a variety of AS events occurred in *SR* genes of *Brassica rapa* under abiotic stresses (Yoon et al., 2018). Recently, an increasing number of studies focused on the detailed functional and regulatory mechanisms of the varied SR transcripts. Numerous SR transcripts contained premature termination codons (PTCs) which might elicit nonsense-mediated mRNA decay (NMD) to regulate the gene expression (McGlincy and Smith, 2008; Palusa and Reddy, 2010). And other SR transcripts showed distinct biological functions, like salt-responsive gene *SR45a* could generate two transcripts SR45a-1a and SR45a-1b, the first of which directly interacted with the cap-binding protein 20 (CBP20), whereas the latter promoted the association of SR45a-1a with CBP20, through the fine-tune regulatory mechanism, it was conducive for the plants to response to salt stress (Li et al., 2021).

*Brassica napus* is an important global oil crop (Chalhoub et al., 2014), which is an allotetraploid species derived from hybridization between *B. rapa* and *Brassica oleracea*. To date, it is unclear how many *SR* genes/transcripts are present in *B. napus* and how they perform their function to affect the oil crop. Now the genome sequences and various transcriptome datasets of *B. napus* are available (Chalhoub et al., 2014; Zhang et al., 2019; Yao et al., 2020), which provide an ample resource to investigate the specific genes at the genome-wide level. In this study, *SR* genes were identified in *B. napus*, the phylogenetic relationship, gene structures, conserved motifs, gene duplications and protein interactions were also analyzed. The transcriptome data from various tissues and environmental stresses were used for the expression patterns and alternative splicing analysis of *SR* genes in *B. napus*. Moreover, genetic variations of *SR* genes in a worldwide core collection germplasm (Tang, 2019) were also investigated, and the association mapping analysis revealed that 12 *SR* genes were candidate genes for agronomic traits in *B. napus*. This study expanded our understanding of *SR* genes in *B. napus* and provided a foundation for further functional studies.

## Materials and Methods

### Identification of *SR* Genes in *Brassica napus*

The genome and annotation information of the *B. napus* cultivar ‘‘*Darmor*-bzh’’ were obtained from the Brassicaceae Database (BRAD)^1^ (Chalhoub et al., 2014). The amino acid sequences of the SR family in *Arabidopsis thaliana* (Kalyna and Barta, 2004) were obtained to build a Hidden Markov Model (HMM), and HMMER3.0 (Mistry et al., 2013) was used to search for *SR* genes in *B. napus* (E value was set to 1e-5). The NCBI Conserved Domain Database^2^ (Lu et al., 2020)and the SMART databases^3^ (Letunic et al., 2020)were used for verification of candidate genes, preserving the ones containing RRM and RS domains. Moreover, ProtParam,^4^ an online software of SWISS-PROT, was used to predict the molecular weights (MW) and isoelectric point (pI) of SR proteins, and CELLO v2.5 (Yu et al., 2006) was used to predict the subcellular location of these proteins.

### Chromosomal Location and Gene Duplication Analysis

The locations of *SR* genes were obtained from the annotation of *B. napus* genome. To identify gene duplication events, BLASTP with the e-value of 1e–10 was used to align the sequence, and MCScanX (Wang et al., 2012) was used to detect the duplication patterns including segmental and tandem duplication. Chromosomal locations and duplication events were visualized using the Circos software (Krzywinski et al., 2009). The ratios of non-synonymous to synonymous substitutions (Ka/Ks) of duplicate gene pairs were counted by ParaAT2.0 (Zhang et al., 2012), which aligned the protein sequence by Muscle (Edgar, 2004) and calculated the Ka/Ks ratio by KaKs_Calculator (Wang et al., 2010).

### Gene Structure, Conserved Motifs, and *cis*-Acting Regulatory Elements Analysis

TBtools (Chen et al., 2020a) and Multiple Expectation Maximization for Motif Elicitation (MEME) (Bailey et al., 2015) were used to display the gene structures and to analyze the conserved motifs in SR proteins. To identify the *cis*-acting regulatory elements of *SR* genes, promoters (2 kb upstream sequences from initiation codon) were extracted and predicted by PlantCARE^5^ (Magali, 2002). The location was displayed by Gene Structure Display Server (GSDS 2.0) (Hu et al., 2015), the amount heatmap was visualized by R.^6^

### Phylogenetic Analysis of SR Family Members

To gain insights into the evolutionary relationships of SR family members, multiple sequence alignments of SR amino acids of *A. thaliana* and *B. napus* were performed using the ClustalW (Larkin, 2007). Phylogenetic trees were generated with the MEGA 11 program using the Neighbor-Joining (NJ) method with 1,000 bootstrap replications (Tamura et al., 2021). The tree was visualized using Evolview^7^ (He et al., 2016).

### Prediction of Protein-Protein Interactions

The Protein-Protein Interactions of *A. thaliana* were downloaded from STRING^8^ (Szklarczyk et al., 2021), the interaction networks of SR proteins in *B. napus* were predicted based on the homologs in *A. thaliana*, and Cytoscape (Shannon et al., 2003) was used to display the interaction. To investigate the involved biological process, genes that interacted with SR proteins were taken out for Gene Ontology and KEGG enrichment analysis by clusterProfiler in R (Yu et al., 2012).

### Expression Analysis of *SR* Genes in *Brassica napus*

Transcriptome data from five tissues (leaf, callus, bud, root, and young silique) and different stress conditions (dehydration, salt, cold and ABA) of *B. napus* cultivar “ZS11” were used in this study (Zhang et al., 2019; Yao et al., 2020), the expression levels of *SR* genes were calculated with Stringtie (Pertea et al., 2015) after alignment with Hisat2 (Kim et al., 2015), and displayed by Pheatmap and UpSet in R. And expression patterns of four genes were showed by TBtools-eFP (Chen et al., 2020a).^9^

### Alternative Splicing Analysis of *SR* Genes in *Brassica napus*

Based on the two sets of transcriptome data, alternative splicing of *SR* genes were also investigated. For the transcript isoform catalog of *B. napus* obtained from Iso-seq (Yao et al., 2020), the AS events were identified by Astalavista (Sylvain and Michael, 2007) and the expression of alternative splicing transcripts of *SR* genes in various tissues were calculated with Stringtie. For the RNA-seq of different stress conditions (Zhang et al., 2019), transcripts were assembled by Stringtie firstly, then the AS events and the expression of alternative splicing transcripts were counted. In order to verify the AS events between paralogous gene pairs, transcriptome data based on EST sequencing of *B. napus* were downloaded and analyzed (Troncoso-Ponce et al., 2011).

### RNA Isolation and qRT-PCR Analysis of *SR* Genes

The seeds of *B. napus* cultivar “ZS11” were germinated and grown in a growth room at 24°C, with a 16/8 h light/dark photoperiod. The leaves and roots were collected from 20-day-old seedlings, while buds were collected from 70-day-old seedings, siliques were harvested 90 days after germination. Samples were immediately stored in liquid nitrogen, and total RNA was extracted from samples using Invitrogen trizol reagent (TRIzol™15596026, United States) according to the manufacturer’s instructions. Total RNA was then reverse-transcribed into complementary DNAs by using the PrimeScript RT reagent Kit With gDNA Eraser (Takara, Japan). The complementary DNAs were used as templates in quantitative reverse-transcription polymerase chain reaction (qRT-PCR) with the gene-specific primers (Supplementary Table 1). qRT-PCR was performed by using SYBR Green Real-time PCR Master Mix (Bio-Rad, United States) in 20 μl reaction mixture and run on CFX96 Real-time PCR system (Bio-Rad, United States). *B. napus* β-actin gene was used as internal standard. All assays were conducted with three biological repeats, and each with three technical repeats. The relative expression level was obtained using the 2^–ΔΔ*Ct*^ method (Livak and Schmittgen, 2001).

### Association Mapping of *SR* Genes in a Natural Population of *Brassica napus*

To understand the natural variations of *SR* genes in *B. napus*, a natural population with 324 worldwide accessions was used in this study (Tang, 2019). SNPs in the gene regions of *SR* genes were extracted and annotated by SnpEff (Cingolani et al., 2012). The agronomic traits including primary flowering time (PFT), full flowering time (FFT1), final flowering time (FFT2), early flowering stage (EFS), late-flowering stage (LFS), flowering period (FP), plant height (PH), branch number (BN), branch height (BH), main inflorescence length (MIL), main inflorescence silique number (MISN), main inflorescence silique density (MISD) were selected (Tang, 2019). With the mixed linear model, a family-based association mapping analysis considering population structure and relative kinship was performed by EMMAX (Kang et al., 2010). The linkage disequilibrium and haplotype blocks were made by LDBlockShow (Dong et al., 2020) and the enriched Gene Ontology terms of interacted proteins were drawn by Cytoscape (Shannon et al., 2003).

## Results

### *SR* Genes Form Seven Subfamilies in *Brassica napus*

After performing HMM search and domain verification, a total of 59 *SR* genes were identified in *B. napus*. The detailed information of each SR was listed in Table 1, including gene ID, genomic location, amino acids (AA) length, isoelectric point (pI), and molecular weight (MW) and so on. The lengths of SR proteins ranged from 130 to 412 AA, with an average length of 293 AA. The pI values were varied from 7.31 to 12.41 and the MW values were varied from 14.92 to 47.02 kDa. According to the prediction of CELLO, it showed that all the SR proteins were located in nuclear.

**TABLE 1:** Characteristics of the *SR* genes in *B. napus* (pI, isoelectric point; MW, molecular weight).

Gene ID	Subfamily	Chromosome	Start	End	Amino acids	pI	MW(kDa)	Exon number	Duplication type	Subcellular Location
BnaA01g14750D	RS	A01	7452346	7455318	338	10.29	39.08	5	WGD or segmental	Nuclear
BnaA03g12870D	RS	A03	5857719	5860659	402	9.97	47.02	6	WGD or segmental	Nuclear
BnaA08g30960D	RS	A08_random	1785553	1788161	348	10.15	40.26	5	WGD or segmental	Nuclear
BnaC01g41640D	RS	C01_random	812746	815639	339	10.29	39.32	5	Dispersed	Nuclear
BnaC03g15710D	RS	C03	7919987	7922439	348	9.87	41.1	5	WGD or segmental	Nuclear
BnaC04g00810D	RS	C04	681412	683420	246	9.87	29.23	5	WGD or segmental	Nuclear
BnaC07g39690D	RS	C07	40437893	40439448	308	9.9	35.26	4	WGD or segmental	Nuclear
BnaC08g31720D	RS	C08	30974005	30975333	276	9.62	31.75	7	WGD or segmental	Nuclear
BnaC08g47240D	RS	C08_random	2078021	2080623	348	10.12	40.27	5	WGD or segmental	Nuclear
BnaA03g00590D	RS2Z	A03	271562	273654	263	10.18	29.47	5	WGD or segmental	Nuclear
BnaA03g17170D	RS2Z	A03	8046335	8048874	316	10.01	35.85	7	WGD or segmental	Nuclear
BnaA05g28890D	RS2Z	A05	20374400	20376670	295	10.03	33.88	6	WGD or segmental	Nuclear
BnaA07g37700D	RS2Z	A07_random	1239429	1241695	291	10.13	32.62	6	WGD or segmental	Nuclear
BnaA09g33780D	RS2Z	A09	24828942	24830905	283	10.13	31.92	6	WGD or segmental	Nuclear
BnaC03g00890D	RS2Z	C03	425179	427495	265	10.29	29.9	5	WGD or segmental	Nuclear
BnaC03g20680D	RS2Z	C03	10976632	10979194	293	10.1	33.37	6	WGD or segmental	Nuclear
BnaC05g43360D	RS2Z	C05	40270977	40273246	287	9.88	33	6	WGD or segmental	Nuclear
BnaC06g14780D	RS2Z	C06	17526686	17529186	288	10.13	32.39	6	WGD or segmental	Nuclear
BnaC08g24530D	RS2Z	C08	26559105	26561152	284	10.07	31.91	6	WGD or segmental	Nuclear
BnaA03g51620D	RSZ	A03	26830836	26832115	199	11.25	22.85	5	WGD or segmental	Nuclear
BnaA04g14520D	RSZ	A04	12206509	12208772	196	11.06	22.03	5	WGD or segmental	Nuclear
BnaA09g54590D	RSZ	A09_random	2700552	2702123	130	9.86	14.92	3	WGD or segmental	Nuclear
BnaAnng28560D	RSZ	Ann_random	32673950	32675820	194	11.22	21.96	6	Dispersed	Nuclear
BnaC04g36280D	RSZ	C04	37798706	37800989	196	11.06	22.03	5	WGD or segmental	Nuclear
BnaC05g19020D	RSZ	C05	12576277	12578087	185	11.28	21.32	4	WGD or segmental	Nuclear
BnaC07g43350D	RSZ	C07	42484671	42485905	197	11.36	22.76	5	WGD or segmental	Nuclear
BnaA09g52820D	SC	A09_random	666454	668617	381	7.38	41.67	9	Dispersed	Nuclear
BnaCnng35170D	SC	Cnn_random	33346654	33348807	366	7.31	40.1	9	Dispersed	Nuclear
BnaCnng52140D	SC	Cnn_random	51593258	51595460	381	7.38	41.66	9	Dispersed	Nuclear
BnaA04g03560D	SCL	A04	2430038	2432468	282	11.37	31.86	7	WGD or segmental	Nuclear
BnaA05g13830D	SCL	A05	8419061	8421417	318	11.77	36.72	5	WGD or segmental	Nuclear
BnaA05g27090D	SCL	A05	19602609	19604747	205	10.75	24.04	5	WGD or segmental	Nuclear
BnaA06g00410D	SCL	A06	214562	217292	284	11.78	32.55	5	WGD or segmental	Nuclear
BnaA08g00560D	SCL	A08	347635	349821	232	11.29	26.56	8	WGD or segmental	Nuclear
BnaC01g37560D	SCL	C01	36838369	36841052	261	11.51	30.38	4	WGD or segmental	Nuclear
BnaC03g70430D	SCL	C03	60111052	60114048	337	11.91	39.05	7	WGD or segmental	Nuclear
BnaC04g25450D	SCL	C04	26579193	26581824	278	11.41	31.53	7	WGD or segmental	Nuclear
BnaC05g41220D	SCL	C05	39032165	39034551	238	10.78	28.34	7	WGD or segmental	Nuclear
BnaC06g07190D	SCL	C06	7792680	7794731	287	11.77	32.59	5	WGD or segmental	Nuclear
BnaC06g10860D	SCL	C06	12913198	12916033	297	11.67	34.02	7	WGD or segmental	Nuclear
BnaCnng00990D	SCL	Cnn_random	1223600	1225715	263	11.54	30.55	4	WGD or segmental	Nuclear
BnaA02g20550D	SR	A02	12929940	12932521	305	11.18	34.06	11	WGD or segmental	Nuclear
BnaA06g15930D	SR	A06	8756159	8759128	289	10.21	32.17	11	WGD or segmental	Nuclear
BnaA06g21030D	SR	A06	14628976	14631673	275	10.55	31.27	12	WGD or segmental	Nuclear
BnaA06g37780D	SR	A06_random	174729	177826	253	9.96	28.94	11	WGD or segmental	Nuclear
BnaA09g00790D	SR	A09	496380	499254	309	11.11	34.38	11	WGD or segmental	Nuclear
BnaC01g26160D	SR	C01	22702333	22705203	282	10.35	31.78	10	WGD or segmental	Nuclear
BnaC02g27300D	SR	C02	25201876	25204492	299	11.01	33.73	11	WGD or segmental	Nuclear
BnaC03g52440D	SR	C03	37261995	37265320	269	10.47	30.55	12	WGD or segmental	Nuclear
BnaC05g06630D	SR	C05	3275956	3279260	317	9.68	36.59	13	WGD or segmental	Nuclear
BnaC07g38480D	SR	C07	39812212	39814540	200	7.67	22.91	9	Dispersed	Nuclear
BnaC08g21130D	SR	C08	23678348	23681381	295	10.32	33.04	11	WGD or segmental	Nuclear
BnaCnng19170D	SR	Cnn_random	17870537	17873473	308	11.05	34.33	11	WGD or segmental	Nuclear
BnaA06g11140D	SR45	A06	5847134	5850099	396	12.41	43.55	12	WGD or segmental	Nuclear
BnaA08g23570D	SR45	A08	16731975	16735088	366	12.34	40.69	11	WGD or segmental	Nuclear
BnaA09g56240D	SR45	A09_random	3650300	3653282	411	12.41	45.25	12	WGD or segmental	Nuclear
BnaC05g12680D	SR45	C05	7414534	7417852	399	12.38	44.09	12	WGD or segmental	Nuclear
BnaC08g16960D	SR45	C08	20742488	20745684	387	12.36	42.48	12	WGD or segmental	Nuclear
BnaC08g38300D	SR45	C08	34660215	34663377	412	12.41	45.29	12	WGD or segmental	Nuclear

**FIGURE 1:**
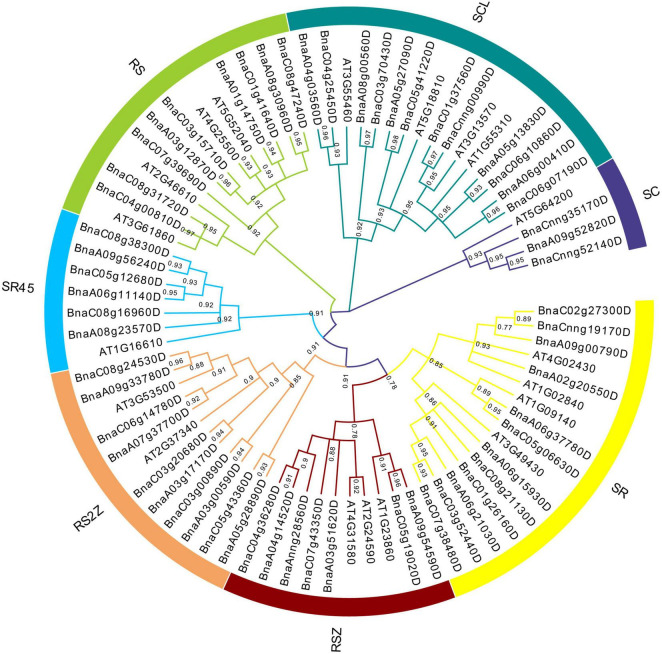
Phylogenetic analysis of SR proteins in *B. napus* and *A. thaliana*. All SR proteins were clustered into seven subfamilies, and each subfamily was represented by a different color.

To understand the evolutionary relationships of *SR* genes between *B. napus* and *A. thaliana*, a phylogenetic tree was constructed based on their protein sequences. Finally, 19 *AtSRs* and 59 *BnSRs* were clustered into seven subfamilies (Figure 1 and Table 1). According to the previous nomenclature system, subfamily SR, SCL, RS2Z, RSZ, RS, SR45, and SC were also used in this study. Subfamily SCL and SR were the largest, each of which included 12 *SR* genes, while subfamily SC was the smallest, with only 3 *SR* genes, and the other subfamily RS2Z, RS, RSZ, and SR45 contained 10, 9, 7, and 6 *SR* genes, respectively.

### Chromosomal Distribution and Gene Duplication of *SR* Genes in *Brassica napus*

**FIGURE 2:**
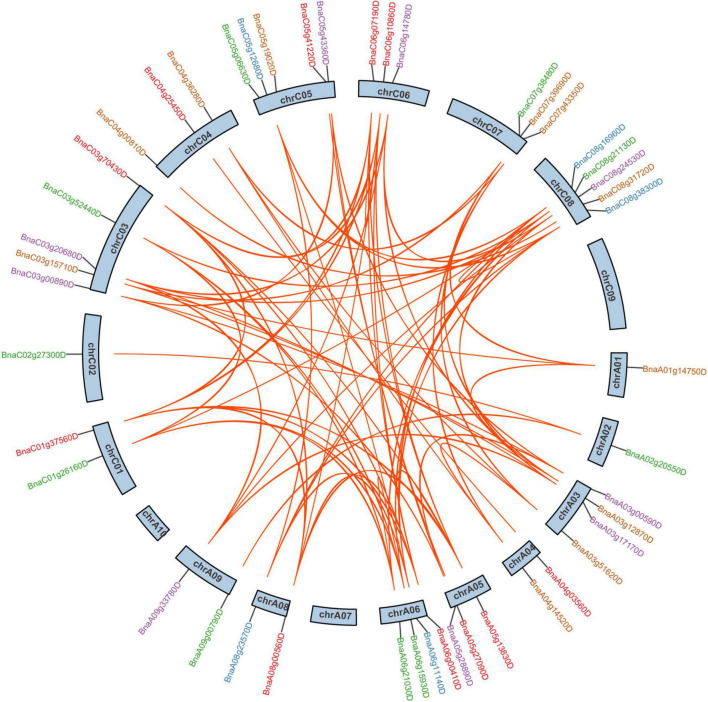
The chromosomal distribution and duplication analysis of *SR* genes in *B. napus*. The locations of all the chromosomal *SR* genes were shown in different chromosomes. The different colors indicated different subfamilies of the *SR* genes. The orange lines highlighted the duplicated *SR* gene pairs.

In *B. napus*, 46 of 59 *SR* genes were unevenly distributed over 19 chromosomes, while the other 13 *SR* genes were assigned to unanchored scaffolds (Figure 2). In total, 26 and 33 *SR* genes were located on the A subgenome and C subgenome, respectively. There were 5 SR, 5 SCL, 5 RS2Z, 4 RSZ, 3 SR45, 3 RS, and 1 SC subfamily genes on A subgenome, with compared to 7 SR, 7 SCL, 5 RS2Z, 3 RSZ, 3 SR45, 6 RS, and 2 SC on C subgenome. Chromosomes C03, C05, and C08 had the most *SR* genes (5 genes per chromosome), while chromosomes A01, A02, and C02 contained only one *SR* gene, respectively, and no *SR* gene was located on chromosomes A07, A10, and C09.

According to BLAST and MCScanX, gene duplication events of the *SR* genes were detected in *B. napus*. In short, all 59 *SR* genes were derived from duplication (Table 1), 89.83% of them (53 *SR* genes) were originated from whole-genome duplication (WGD) or segmental duplications, while the other 6 *SR* genes resulted from dispersed duplications. Moreover, there were 91 paralogous gene pairs in *B. napus* (Figure 2 and Supplementary Table 2), 15 of them occurred in the A subgenome, 21 of them took place in the C subgenome, and the other 55 duplication events occurred between the A and C subgenome. To estimate the selection mode of *SR* genes in *B. napus*, the ratios of non-synonymous to synonymous substitutions (Ka/Ks) for paralogous gene pairs were calculated. Generally, Ka/Ks > 1 means positive selection, Ka/Ks = 1 means neutral selection, and Ka/Ks < 1 represents purifying selection. In this work, Ka/Ks ratios of all the paralogous gene pairs were less than 1, suggesting that *SR* genes were under purifying selection (Supplementary Table 2).

### Gene Structure, Conserved Motifs, and *cis-*Acting Regulatory Elements Analysis of *SR* Genes in *Brassica napus*

**FIGURE 3:**
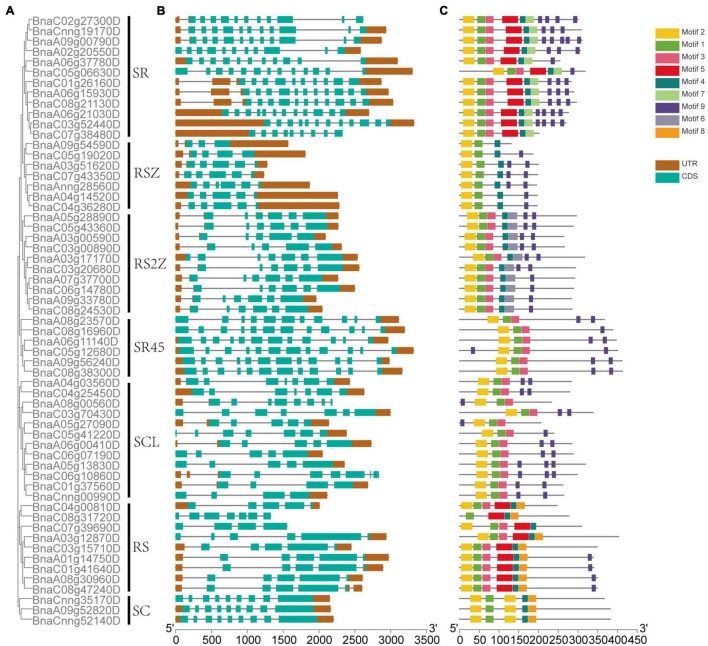
The phylogenetic relationship, gene structure, and conserved motifs of *SR* genes in *B. napus*. **(A)** The phylogenetic relationships of 59 SR proteins based on the NJ method. **(B)** Gene structures of the *SR* genes. Dark brown boxes represented UTR, and indigo boxes represented CDS. **(C)** The motif composition of SR proteins. Numbers 1–9 were displayed in different colored boxes.

The exon-intron structure of 59 *SR* genes in seven subfamilies was displayed (Figures 3A,B), On average, each gene included 7 exons, but the exon numbers differed widely, ranging from 3 to 13, and different subfamilies exhibited different exon numbers. While the genes in the same subfamily tended to possess similar gene structures, for example, in subfamily SC, all the *SR* genes had 9 exons, and in subfamily SR45, all the *SR* genes contained 12 exons except *BnaA08g23570D*.

In total, 9 conserved motifs were identified in 59 *SR* genes (Figure 3C). All the *SR* genes contained motif 1 and motif 2 except *BnaC08g31720D*, which lacked motif 1. All the *SR* genes possessed motif 9, except those in subfamily SC. Apparently, the motif structures of distinct subfamilies varied. For example, the pattern of subfamily SC was motif 2-1-2-4-8, while subfamily RS2Z was motif 2-1-3-4-6-9. And some subfamilies had a few specific motifs, like motif 7 was unique to subfamily SR, motif 8 only existed in subfamily RS2Z.

**FIGURE 4:**
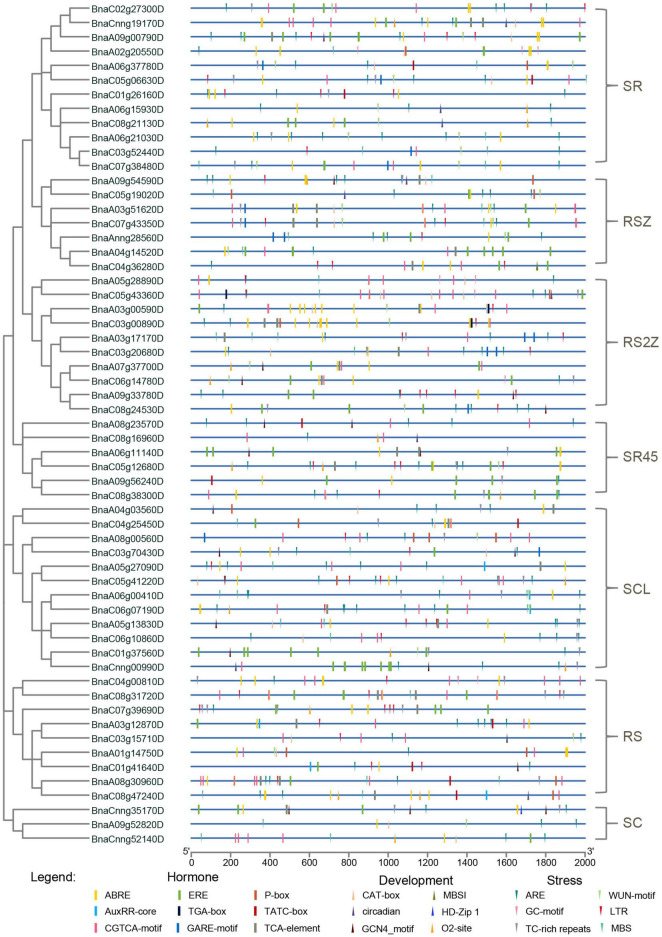
*Cis*-acting regulatory elements identified in promoters of *SR* genes in *B. napus*. Boxes indicated hormone-related elements, up-triangles indicated development-related elements, and down-triangles indicated stress-related elements. Different colors indicated different elements.

Promoter regions were found to be critical for gene expression (Oudelaar and Higgs, 2021), so *cis*-acting regulatory elements in these regions were investigated for *SR* genes. *Cis*-acting regulatory elements related to stress, hormone and development (ranging from 5 to 23) were detected in promoters of *SR* genes (Figure 4, Supplementary Figure 1, and Supplementary Table 3). The majority of *SR* genes (56/59, 94.92%) contained ARE elements, which is essential for anaerobic induction. Moreover, stress-responsive elements such as TC-rich repeats (involved in defense and stress responsiveness, 33/59, 55.93%), LTR (involved in low-temperature responsiveness, 33/59, 55.93%) and MBS (involved in drought-inducibility, 29/59, 49.15%) were also common in promoters of *SR* genes. Hormone-responsive elements like ABRE (involved in the abscisic acid responsiveness), CGTCA-motif (involved in the MeJA-responsiveness) and ERE (involved in the ethylene responsiveness) existed in most promoters of *SR* genes. In terms of development-related elements, CAT-box (24/59, 40.68%), which is related to meristem expression, was most frequently observed in the promoters of *SR* genes. The results indicated that many *SR* genes in *B. napus* were responsible for plant growth and stress response.

### Predicted Protein Interactions of *SR* Proteins in *Brassica napus*

**FIGURE 5:**
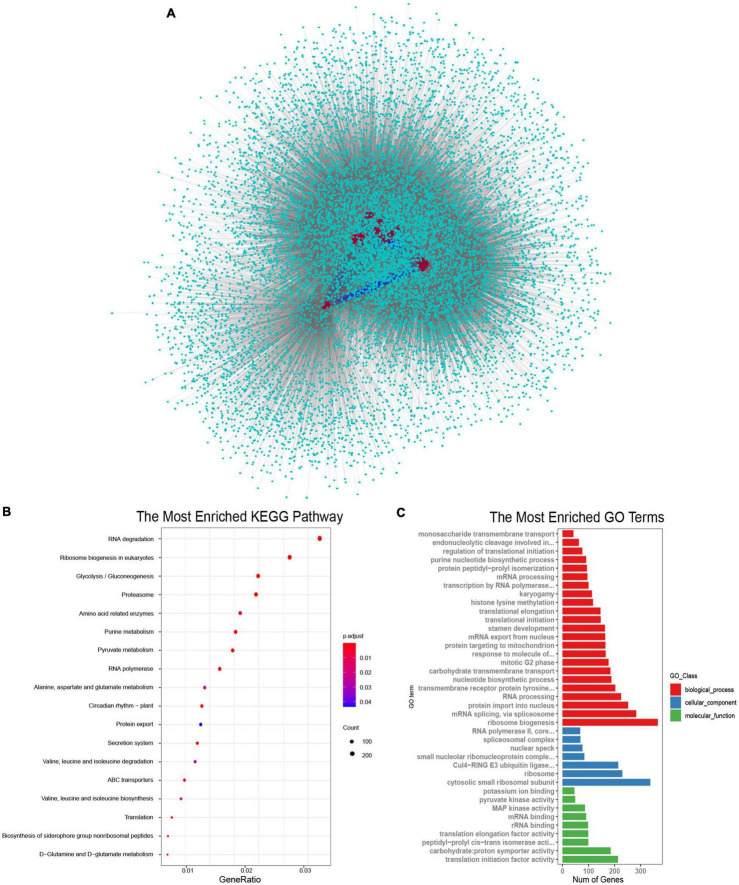
Proteins interacted with SR proteins in *B. napus*. **(A)** Protein-protein interaction network of SR proteins in *B. napus*. The red circles represented the SR proteins, the indigo circles represented proteins that interacted with SR proteins. The blue lines represented the interaction between SR proteins, and the gray lines represented the interaction between SR proteins and other proteins. **(B)** KEGG pathway enrichment analysis of proteins interacted with SR proteins. **(C)** Gene Ontology enrichment analysis of proteins interacted with SR proteins.

SR proteins were the key components of the spliceosome and they always interacted with other proteins to perform their functions (Shepard and Hertel, 2009; Will and Luhrmann, 2010). To understand the biological processes involved by SR proteins in *B. napus*, interaction networks were predicted according to known protein interactions in Arabidopsis. The homologous proteins of 59 BnSR proteins interacted with 3,528 proteins in Arabidopsis, which were homologous to 13,591 proteins in *B. napus* (Figure 5A). It demonstrated that SR proteins were the core nodes in the network, most SR proteins interacted with each other, meanwhile, they also interacted with other proteins to participate in different biological processes. KEGG enrichment analysis showed these interacted proteins were involved in a variety of processes including RNA degradation, ribosome biogenesis, RNA polymerase, proteasome, circadian rhythm, and so on (Figure 5B and Supplementary Table 4). Gene Ontology enrichment analysis (Figure 5C and Supplementary Table 5) showed that ribosome biogenesis, mRNA splicing and protein import into the nucleus were the significantly enriched terms in the biological process category. While the terms including cytosolic small ribosomal subunit and ribosome in the cellular component category were highly enriched, and in the molecular function category, translation initiation factor activity, proton symporter activity and RNA binding were significantly enriched. Protein-protein interactions analysis showed that SR proteins played important roles in the regulation of the whole lifecycle of mRNA, from synthesis to decay.

### Various Expression Patterns of *SR* Genes in Different Tissues and Under Abiotic Stresses in *Brassica napus*

**FIGURE 6:**
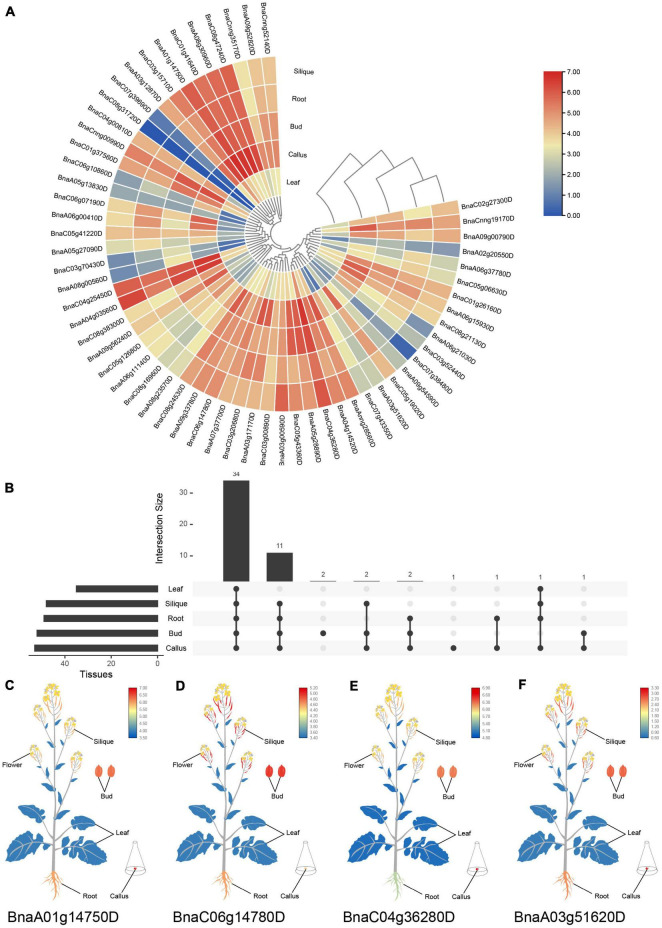
Expression profiles of *SR* genes in different tissues of *B. napus*. **(A)** Heatmap representation of 59 *SR* genes in different tissues. **(B)** Number of *SR* genes that were expressed in various tissues. **(C–F)** The expression patterns of four selected *SR* genes in *B. napus* plants. Expression data were processed with log_2_ normalization. The color scale represented relative expression levels from low (blue color) to high (red color).

To predict the potential functions of *SR* genes, expression patterns based on RNA-Seq of five tissues in *B. napus* cultivar “ZS11” (Yao et al., 2020) were displayed in Figure 6. *SR* genes showed different expression patterns among the five tissues. The expression profiles of *SR* genes in the silique and root displayed similar patterns. Almost all the *SR* genes were expressed highly in bud, root, silique and callus, but lowly in leaf (Figure 6A). There was 34 *SR* genes expressed in all of the five tissues based on the threshold value (FPKM > 5), and some of the *SR* genes were tissue-specific or preferential expression (Figure 6B). Like *BnaA01g14750D* showed the highest expression in callus (Figure 6C), and *BnaC06g14780D* expressed at a high level in silique and bud (Figure 6D), nevertheless, both of them expressed lowly in leaf. Meanwhile, a few *SR* genes expressed highly in callus and lowly in silique. And two *SR* genes (*BnaC08g31720D* and *BnaC07g39690D*) barely expressed in these five tissues.

**FIGURE 7:**
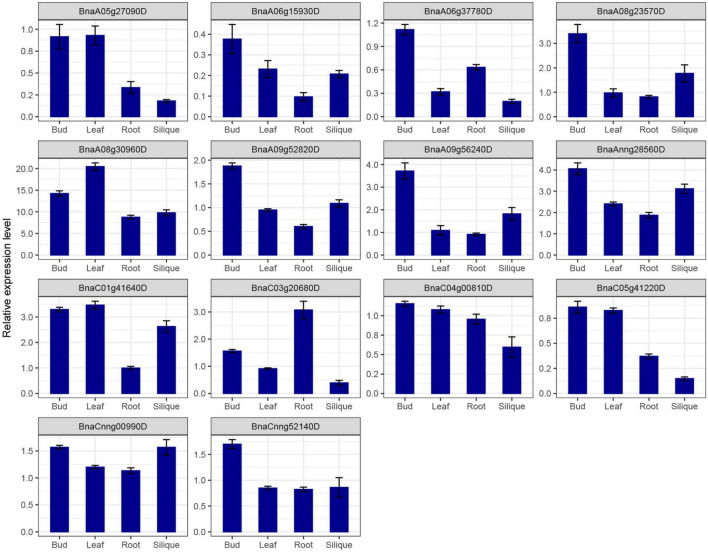
qRT-PCR expression analysis of 14 *SR* genes in different tissues of *B. napus*. The error bars represented the standard error of the means of three replicates.

*SR* genes in subfamily RS2Z, SR45, and SC showed similar expression patterns, paralogous gene pairs in these subfamilies also owned similar expression patterns, like *BnaA09g33780D*/*BnaC06g14780D* in RS2Z, *BnaA06g11140D*/*BnaC05g12680D* in SR45. Nevertheless, in other subfamilies, different patterns were observed, for example, paralogous gene pairs (*BnaA04g03560D*/*BnaC04g25450D*) in subfamily SCL expressed at the same pattern, while in subfamily RS *BnaC08g31720D* barely expressed in five tissues, its paralogous gene *BnaC04g00810D* expressed at a high level in callus, bud, root and silique, and in subfamily RSZ, *BnaC04g36280D* and its paralogous gene *BnaA04g14520D* expressed at a high level in each tissue (Figure 6E), but their paralogous gene *BnaA03g51620D* and *BnaC07g43350D* weakly expressed (Figure 6F). Moreover, 14 *SR* genes from different subfamilies were selected for qRT-PCR analysis (Figure 7 and Supplementary Table 1), similarly, most of these genes expressed higher in bud, and the expression patterns of two genes (*BnaA09g52820D* and *BnaCnng52140D*) from subfamily SC were almost the same, while in subfamily SCL, *BnaCnng00990D* showed different expression patterns with *BnaA05g27090D* and *BnaC05g41220D*.

**FIGURE 8:**
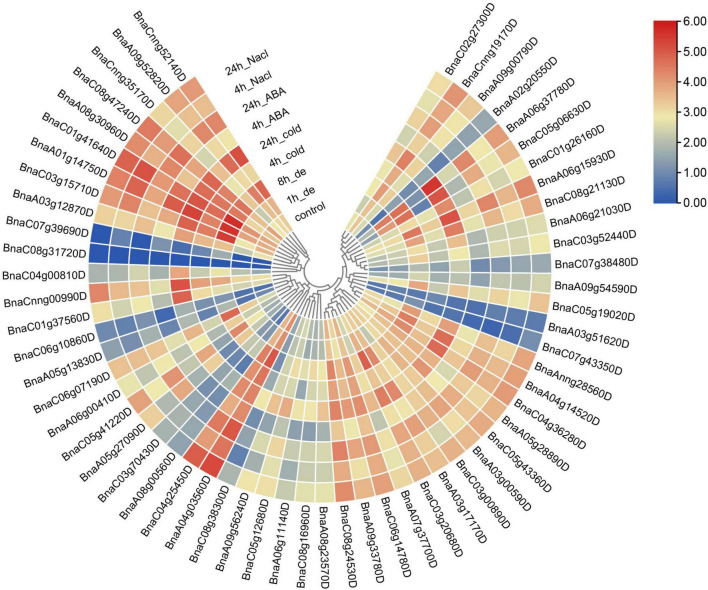
Expression profiles of *SR* genes under abiotic stress conditions. Expression data were processed with log_2_ normalization. The color scale represented relative expression levels from low (blue color) to high (red color).

In spite of expression patterns in various tissues were investigated, the expression profiles of *SR* genes under different abiotic stresses were also analyzed. In this study, RNA-Seq data of samples from different abiotic treatments including cold, drought, salinity, ABA induction (Zhang et al., 2019) were utilized to analyze the expression pattern of *SR* genes in *B. napus* (Figure 8). Obviously, all the *SR* genes expressed higher after the treatment of abiotic stresses except those unexpressed or low-expressed genes. The expression of *BnaC07g39690D* was apparently up-regulated under dehydration stress. The expression of *BnaC05g06630D* dramatically increased under ABA induction as well as cold and salt stress, and it was noticed that elements response to these stresses (ABRE, LTR, and TC-rich repeats) were enriched in its promoter. All the *SR* genes expressed at a higher level in both subfamily RS2Z and subfamily SC, but in other subfamilies, different expression patterns were observed, especially for some paralogous gene pairs, like *BnaC03g15710D*/*BnaC07g39690D*, *BnaC04g00810D*/*BnaC08g31720D*, and *BnaA02g20550D*/ *BnaA09g00790D*, coincidentally, these gene pairs also showed different patterns in various tissues, which suggested they were differentiated into different directions, and the low-expressed genes like *BnaC07g39690D*, *BnaC04g00810D* and *BnaA02g20550D* may become pseudogenes.

### Alternative Splicing of *SR* Genes Is Widespread in *Brassica napus*

**FIGURE 9:**
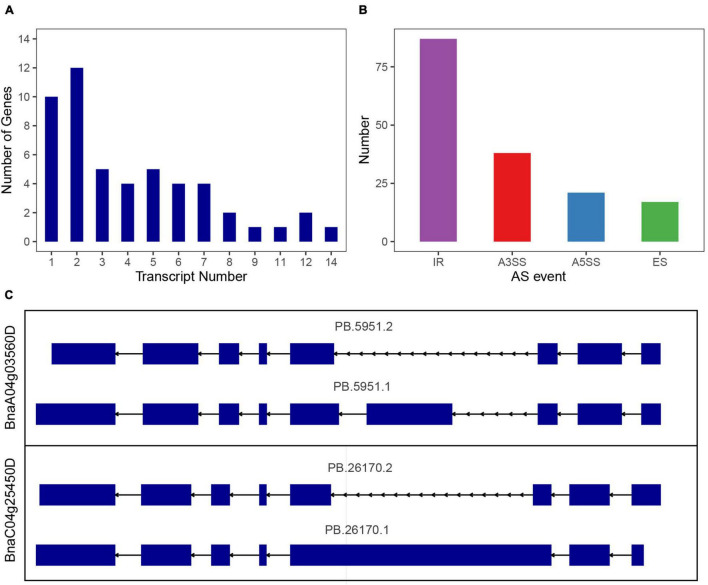
Splicing profiles of *SR* genes in *B. napus* based on Iso-Seq data. **(A)** The numbers of *SR* genes that produced one or more transcripts from Iso-Seq data. **(B)** Classification of AS events. IR, intron retention; A3SS, alternative 3′ splice site; A5SS, alternative 5′ splice site; ES, exon skipping. **(C)** Different splicing patterns of a paralogous gene pair.

In Arabidopsis, maize and sorghum, most of the *SR* genes could be alternatively spliced, in order to investigate the alternative splicing (AS) of *SR* genes in *B. napus*, we used the dataset from Pacbio Iso-Seq technique, which could directly detect the existed mRNA and provide full-length transcripts. Based on Iso-Seq of *B. napus* cultivar “ZS11” (Yao et al., 2020), 51 of 59 *SR* genes were detected in this dataset, and 41 *SR* genes were alternative spliced, yielding 206 transcripts, an average of 5 transcripts for each gene (Figure 9A and Supplementary Table 6). As to each subfamily, *SR* genes in subfamily RS owned the most transcripts per gene (average 6.4 transcripts), whereas *SR* genes in subfamily SC contained the least transcripts, only 1.7 transcripts per gene, and the other subfamily RS, SR45, RS2Z, SCL, and RSZ contained 6.2, 4.3, 4.3, 2, and 1.8 transcripts, respectively. In the multi-exon *SR* genes, a total of 163 AS events were discovered, intron retention (IR) was the most one (87), followed by alternative 3′ splice site (A3SS, 38), alternative 5′ splice site (A5SS, 21) and exon skipping (ES, 17) (Figure 9B). Subfamily RS had 51 AS events (IR-29, A3SS-8, A5SS-9, ES-5), which was the most and consistent with its most transcripts. While the fewer transcripts in subfamily RSZ and SC contained fewer AS events. Most of the paralogous gene pairs displayed distinct splicing patterns, the first one was the transcripts number varied between paralogous gene pairs, like 2 transcripts of *BnaA06g11140D* vs. 4 transcripts of *BnaC05g12680D*, and 8 transcripts of *BnaA03g17170D* vs. 3 transcripts of *BnaA07g37700D*, the second one was the AS events varied between paralogous gene pairs, both *BnaA04g03560D* and *BnaC04g25450D* had 2 transcripts, but the identified AS events were different (Figure 9C). To verify the AS events, the detailed alignment information was displayed, and it showed that a small number of reads could span the splice sites (Supplementary Figure 2). Moreover, EST dataset was also used to blast against the alternative splicing transcripts, and the results revealed that the different AS events really existed (Supplementary Table 7). To find out the expression patterns of transcripts in various tissues, the expression levels of all the transcripts of *SR* genes were also counted (Supplementary Figure 3), and it showed that only a fraction of them expressed higher in these tissues, for paralogous gene pair *BnaA04g03560D*/*BnaC04g25450D*, the expression patterns of their transcripts were also different.

Moreover, in the RNA-seq of abiotic stresses, the short reads were assemblied to predict the splicing profiles (Supplementary Figure 4), finally 124 transcripts were detected in 46 genes, and 61 AS events were identified. In this dataset, IR was not the most prevalent AS type, instead, A3SS was more prevalent. Five transcripts of *BnaA06g37780D*, *BnaC05g06630D*, and *BnaA01g14750D* were obviously induced by all four stresses, and the increment was obvious as the treatment time increased (Supplementary Figure 4), indicating that they were the responsible splicing factors responding to abiotic stress in *B. napus*.

### Genetic Effects of *SR* Genes on Agronomic Traits of *Brassica napus*

To investigate the genetic variations of *SR* genes, SNPs were identified in a natural population containing 324 accessions collected from worldwide countries (Supplementary Table 8; Tang, 2019). Averagely, each *SR* gene contained 43 SNPs, lower than the whole genome level (94 SNPs in each gene). In consideration of genome size, we calculated the average SNP number of each kilobase (kb), all the *SR* genes were 17 SNPs/kb, while the whole genome level was 11 SNPs/kb. The SNP density of *SR* genes in the A subgenome (22 SNPs/kb) was slightly higher than the C subgenome (13 SNPs/kb). Moreover, the SNP density varied in different subfamilies, like subfamily SR45 had the most, with an average of 90 SNPs, followed by RSZ (41 SNPs) and SCL (39 SNPs), while SC had the fewest (only 29 SNPs). We also examined the genetic variations of paralogous gene pairs, there were 97 SNPs in *BnaA09g00790D*, but none in its paralogous gene *BnaCnng19170D*, while paralogous gene pairs *BnaC04g00810D*/*BnaC08g31720D*, had 49 and 5 SNPs, respectively. On the whole, most paralogous gene pairs exhibited unequal variations. Finally, SNP annotation showed that 658 SNPs occurred in exon regions and 194 SNPs in 39 *SR* genes resulted in missense mutations.

**FIGURE 10:**
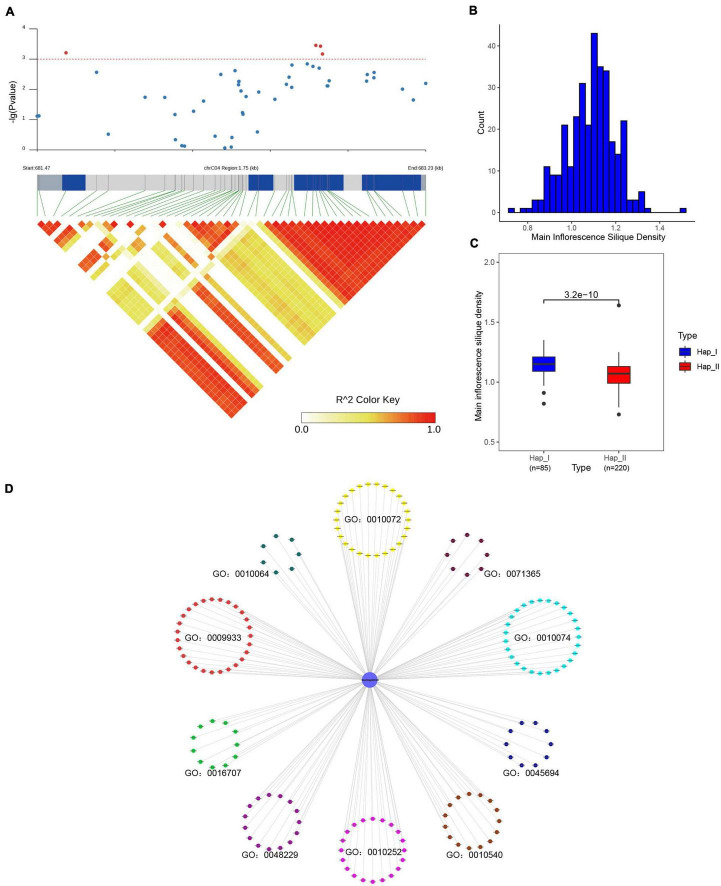
Association mapping analysis of *BnaC04g00810D* in 324 core collections of *B. napus* germplasm. **(A)** *BnaC04g00810D* was significantly associated with main inflorescence silique density. **(B)** The distribution of main inflorescence silique density. **(C)** Comparison of main inflorescence silique density between the two haplotypes based on the most significantly associated SNP of *BnaC04g00810D*. **(D)** The enriched Gene Ontology terms of interacted proteins of *BnaC04g00810D*.

For *SR* genes were the fundamental regulators in pre-mRNA processing, it could affect various physiological processes, and finally result in diverse phenotype (Shepard and Hertel, 2009; Reddy and Shad Ali, 2011). In order to study the impact of *SR* genes on agronomic traits in *B. napus*, the association mapping analysis was conducted for 12 agronomic traits. In total, 49 SNPs (corresponding to 12 *SR* genes, Supplementary Table 8) located on A03, A05, A09, C03, C04, C05, C06, C07 and unanchored scaffolds were significantly associated with one or more agronomic traits (*p* < 0.001). *BnaC04g00810D* was significantly associated with main inflorescence silique density (Figures 10A,B), and the missense mutation in the coding sequence changed the arginine to histidine (305G > A). According to the genotype, two groups were divided and the main inflorescence silique density was significantly different based on the *t*-test (*p* < 3.2e–10) (Figure 10C). The interacted proteins of *BnaC04g00810D* were analyzed, they were not only enriched in mRNA splicing and spliceosome, but also enriched in the maintenance of meristem identity (GO:0010074), regulation of embryo sac egg cell differentiation (GO:0045694), meristem structural organization (GO:0009933), primary shoot apical meristem specification (GO:0010072), embryonic shoot morphogenesis (GO:0010064), gibberellin 3-beta-dioxygenase activity (GO:0016707), auxin homeostasis (GO:0010252), basipetal auxin transport (GO:0010540), cellular response to auxin stimulus (GO:0071365) (Figure 10D). As we knew, gibberellins (GAs) could promote stem elongation and floral development during bolting (Olszewski et al., 2002), auxin biosynthesis and transport played an important role in floral meristem initiation and inflorescence organization (Teo et al., 2014). All these processes were related with the regulation of endogenous hormone and the development of meristem/gametophyte, which could affect the silique density (Ren et al., 2018). The interacted proteins of *BnaC04g00810D* took part in these processes, like GA3OX1/2/4 in GO:0016707 were responsible for the last step of the biosynthetic of active GAs (Williams et al., 1998), ABCB19 in GO:0010540 mediated polar auxin transport (Wu et al., 2016), and GAF1 was involved in female gametophyte development (Zhu et al., 2016). Therefore, it was speculated that *BnaC04g00810D* also participated in the above processes through interacting with related proteins and might be an important candidate gene for silique density in *B. napus*. Moreover, *BnaA03g12870D* was significantly associated with flowering time and branch number, whereas *BnaC03g20680D* was significantly associated with the flowering period (Supplementary Figure 5), and the involved processes of their interacted proteins were also enriched in meristem structural organization, regulation of flower development and so on. Overall, the results suggested that sequence variations of *SR* genes could affect the development of *B. napus* and, ultimately influence the important agronomic traits.

## Discussion

Alternative splicing plays important role in the plant growth and development process, especially enhancing the adaptability of plants under stress conditions (Black, 2003; Palusa et al., 2007). Splicing factors are essential for the execution and regulation of splicing. Among them, SR proteins are the prominent factors involved in the assembly of spliceosomes, recognition and splicing of pre-mRNAs (Zahler et al., 1992). Recently, SR proteins in many plants have been studied at the genome-wide level to understand their evolution and function (Kalyna and Barta, 2004; Isshiki et al., 2006; Richardson et al., 2011; Chen et al., 2019, 2020b; Gu et al., 2020). In this study, 59 *SR* genes were identified and characterized in *B. napus*. A systematical analysis of *SR* genes including chromosomal locations, gene structures, conserved motifs, phylogenetic relationships, and protein-protein interactions was performed. Moreover, the expression patterns and AS types of *SR* genes in various tissues and stresses were analyzed. Variations in *SR* gene sequences and the association mapping analysis based on various agronomic traits were also performed to detect the relationship between *SR* genes and the final phenotype in *B. napus*.

After divergence from Arabidopsis lineage, the genus *Brassica* underwent a genome triplication event that occurred 13 million years ago, then interspecific hybridization between *B. rapa* and *B. oleracea* formed the allotetraploid *B. napus* (Allender and King, 2010). All the genes in *B. napus* expanded during its evolution and formation (Chalhoub et al., 2014). Many studies had shown that whole-genome duplication (WGD) and segmental duplications were the key factors to produce duplicated genes and result in the expansion of gene families (Ma et al., 2017; Wu et al., 2018; Zhu et al., 2020), as well as observed in *SR* genes in this study. Based on the effect of two recent duplication events, six homologs for each Arabidopsis gene were expected to present in *B. napus*, but we only found 59 *SR* genes in *B. napus* (about threefold of AtSRs), which indicated that gene loss happened (Albalat and Canestro, 2016). And the distribution of *SR* genes in the A and C subgenome implied the gene loss is asymmetrical, which is consistent with the genome level (Chalhoub et al., 2014). According to the Ka/Ks ratios of paralogous gene pairs, it is suggested that purifying selection played an important role in the evolution of *SR* genes in *B. napus*.

In plants, *SR* gene family had been investigated in Arabidopsis, rice, maize, wheat, tomato, cassava, and so on (Kalyna and Barta, 2004; Isshiki et al., 2006; Richardson et al., 2011; Yoon et al., 2018; Chen et al., 2019, 2020b; Gu et al., 2020; Rosenkranz et al., 2021). Most of the *SR* genes were divided into five to seven subfamilies according to the domain sequence or the whole sequence, likewise, 59 *SR* genes in *B. napus* were also classified into seven subfamilies. The proportion of plant-specific subfamily members in *B. napus* (31/59, 52.54%) was similar to that of other plants (Chen et al., 2019). Most genes in the same subfamily shared similar gene structures, conserved motifs, but the *cis*-acting regulatory elements in promoters emerged a big difference, which would affect the expression patterns (Zou et al., 2011; Oudelaar and Higgs, 2021). In the RNA-seq of various tissues, *SR* genes expressed obviously lower in leaf in comparison with bud, root, silique and callus, which was probably due to more complex splicing events in differentiated organs than mature organs, similarly, it had been proved that many *SR* genes expressed highly in early stages of fruit growth and development in tomato, which indicated a higher demand for factors to regulate pre-mRNA processing during cell expansion in immature green fruits (Rosenkranz et al., 2021). Various expression patterns of duplicated genes were also observed in this study, and it had been proved as one common way to lead to pseudogenization, neofunctionalization, or subfunctionalization in polyploids (Chaudhary et al., 2009). The lifestyle of plants is sessile, which is different from animals, environmental factors such as light, temperature, water or soil characteristics strongly influence their growth and development. As a result, plants have intelligently evolved various strategies for fleetly responding to changes (Meena et al., 2017). The diverse *cis*-acting regulatory elements in the promoter regions of different *SR* genes indicated their expression could be induced by hormones or abiotic stress. The different types, copy numbers and combinations of *cis*-acting regulatory elements predicted the diversity of *SR* genes expression patterns and flexibility in response to different stresses. Under environmental stress or hormone induction, the expression patterns of most *SR* genes changed. Expression of *BnaA06g37780D* and *BnaC05g06630D* increased with the treatment of cold, drought, salinity and ABA, and it had been verified that its orthologous gene *AtSR30* was up-regulated by salinity stress (Tanabe et al., 2007).

Transcription is a flexible mechanism, which not only alters the gene expression but also could create diverse transcripts (Herbert and Rich, 1999). With the development of sequencing technology, it is possible to provide full-length transcripts by Iso-Seq directly (Abdel-Ghany et al., 2016; Wang et al., 2017), avoiding sequence assembly by short reads from RNA-seq. In the Iso-Seq data of the five tissues (Yao et al., 2020), 41 *SR* genes were alternatively spliced to produce 206 transcripts, which increased the transcriptome complexity greatly. If datasets from other various tissues and treatments were obtained, it was speculated that the amounts of SR transcripts were astounding in *B. napus*. AS not only regulated the gene expression, but also could cause neofunctionalization or subfunctionalization between paralogous genes (Zhang et al., 2009). Here we found diverse AS patterns that occurred in the paralogous gene pairs, this result supplied a clue for further functional study which would focus on the different transcripts of *SR* genes. Furthermore, *SR* genes generated a variety of transcripts by alternative splicing in response to abiotic stress. In Arabidopsis, it had been proved that the alternatively spliced transcripts of several *SR* genes were directly associated with plants’ ability to adapt to different environmental stresses (Palusa et al., 2007; Rauch et al., 2014). Similarly, 21 SR transcripts were detected under salt stress in cassava, which indicated these transcripts might participate in the biological process induced by salt (Gu et al., 2020). In this study, five transcripts from three *SR* genes obviously increased their expression after prolonged treatments of four different stresses. However, further research is required to determine the precise function and regulatory mechanisms of these SR transcripts in response to abiotic stress.

Sequence variations of *SR* genes were investigated in a natural population of *B. napus* (Tang, 2019), the SNP density in *SR* genes was higher than the average level of the genome, implying that abundant variations have accumulated in the evolution of *SR* gene family. The greater SNP prevalence of *SR* genes in the A subgenome was consistent with other gene families such as *GATAs* in a core collection of *B. napus* (Zhu et al., 2020). For genes in polyploids, after predicting function through their orthologs, to distinguish the one which performs function among several paralogous genes is another question. One way is to verify the function of paralogous genes one by one through traditional transgenic analysis, another way is with the aid of association mapping analysis. Typically, changes between paralogous gene pairs were distinct, leading to pseudogenization, neofunctionalization or subfunctionalization (Schiessl et al., 2017). For example, in contrast to *Bn-CLG1C*, a dominant point mutation in *Bn-CLG1A* led to cleistogamy in *B. napus*, which was regarded as a gain-of-function semi-dominant mutation (Lu et al., 2012). A single “C-T” mutation in the coding sequence of *BnaA03.CHL*H hindered chloroplast development, resulting in yellow-virescent leaf, while *BnaC03.CHLH* maintained the virescent color of the leaf (Zhao et al., 2020). In this study, 194 missense mutations could introduce various divergences of *SR* genes in *B. napus*. For paralogous gene pairs *BnaC04g00810D*/*BnaC08g31720D*, the expressions of *BnaC04g00810D* in tissues were higher than *BnaC08g31720D*, the missense mutation in the coding sequence of *BnaC04g00810D* changed the arginine to histidine, the association analysis and enriched processes of interacted proteins indicated that it was candidate gene for regulating silique density in *B. napus*. In previous studies, over-expression or transgenic analysis had proved that *SR* genes could affect the development and morphology in Arabidopsis (Kalyna et al., 2003; Ali et al., 2007), although none of the *SR* genes were studied by experimental analysis in *B. napus*, the association mapping analysis performed in this study could provide a useful clue for understanding the effect of *SR* genes on final phenotype and supply candidate genes for further improving agronomic traits in *B. napus*.

## Conclusion

In this study, a comprehensive genome-wide identification and characterization of *SR* genes in *B. napus* were conducted. In total, 59 *SR* genes were identified and classified into seven subfamilies. Genes belonging to the same subfamily shared similar gene structures and motifs. *Cis*-acting regulatory elements in the promoters of *SR* genes and expression patterns in various tissues and environmental stresses revealed that they played important roles in development and stress responses. Transcriptome datasets from Pacbio/Illumina platforms showed that alternative splicing of *SR* genes was widespread in *B. napus* and the majority of paralogous gene pairs displayed different splicing patterns. Protein-protein interaction analysis showed that *SR* genes were involved in the whole lifecycle of mRNA, from synthesis to decay. Furthermore, genetic variations in *SR* genes were also investigated, and the association mapping results indicated that 12 *SR* genes were candidate genes for regulating specific agronomic traits. In summary, these findings provide elaborate information about *SR* genes in *B. napus* and may serve as a platform for further functional studies and genetic improvement of agronomic traits in *B. napus*.

## Data Availability Statement

The original contributions presented in the study are included in the article/Supplementary Material, further inquiries can be directed to the corresponding authors.

## Author Contributions

MX, CT, and SL designed the research. MX, RZ, ZB, CZ, and LY performed the experiments. MX, RZ, FG, XC, JH, and YuL analyzed the data. MX, CT, and YaL wrote and revised the manuscript. All authors have read and approved the current version of the manuscript.

## Conflict of Interest

The authors declare that the research was conducted in the absence of any commercial or financial relationships that could be construed as a potential conflict of interest.

## Publisher’s Note

All claims expressed in this article are solely those of the authors and do not necessarily represent those of their affiliated organizations, or those of the publisher, the editors and the reviewers. Any product that may be evaluated in this article, or claim that may be made by its manufacturer, is not guaranteed or endorsed by the publisher.

## Footnotes

1
http://brassicadb.cn/

2
https://www.ncbi.nlm.nih.gov/Structure/cdd/wrpsb.cgi

3
http://smart.embl.de/

4
https://web.expasy.org/protparam/

5
http://bioinformatics.psb.ugent.be/webtools/plantcare/html/

6
https://cran.r-project.org/

7
https://www.evolgenius.info/evolview/

8
https://www.string-db.org/

9
http://yanglab.hzau.edu.cn/BnTIR/eFP

## Supplementary Material

Supplementary Figure 1 Amount of *cis*-acting regulatory elements in promoters of *SR* genes in *B. napus*. Elements numbers were processed with log_2_ normalization. The color scale represented amounts from low (blue color) to high (red color).

Supplementary Figure 2 The alignment information of *BnaA04g03560D* and *BnaC04g25450D.*

Supplementary Figure 3 Heatmap representation of transcripts of *SR* genes in different tissues. Expression data were processed with log_2_ normalization. The color scale represented relative expression levels from low (blue color) to high (red color).

Supplementary Figure 4 Splicing profiles of *SR* genes in *B. napus* under abiotic stress condition. **(A)** Distribution of genes that produced one or more transcripts from RNA-Seq data. **(B)** Classification of AS events from RNA-Seq data. IR, intron retention; A3SS, alternative 3′ splice site; A5SS, alternative 5′ splice site; ES, exon skipping. **(C)** Five transcripts were obviously induced by all four stresses. Expression data were processed with log_2_ normalization. The color scale represented relative expression levels from low (blue color) to high (red color).

Supplementary Figure 5 Association mapping analysis of *SR* genes in 324 core collections of *B. napus* germplasm. **(A,B)** *BnaA03g12870D* was significantly associated with primary flowering time. **(C,D)** *BnaA03g12870D* was significantly associated with branch number. **(E,F)** *BnaC03g20680D* was significantly associated with the flowering period.
