# Supplementary figures and images for: Genome-Wide Characterization of Serine/Arginine-Rich Gene Family and Its Genetic Effects on Agronomic Traits of Brassica napus
Source: Front Plant Sci. 2022 Feb 16;13:829668. doi: 10.3389/fpls.2022.829668 (PMC8889041; doi:10.3389/fpls.2022.829668)

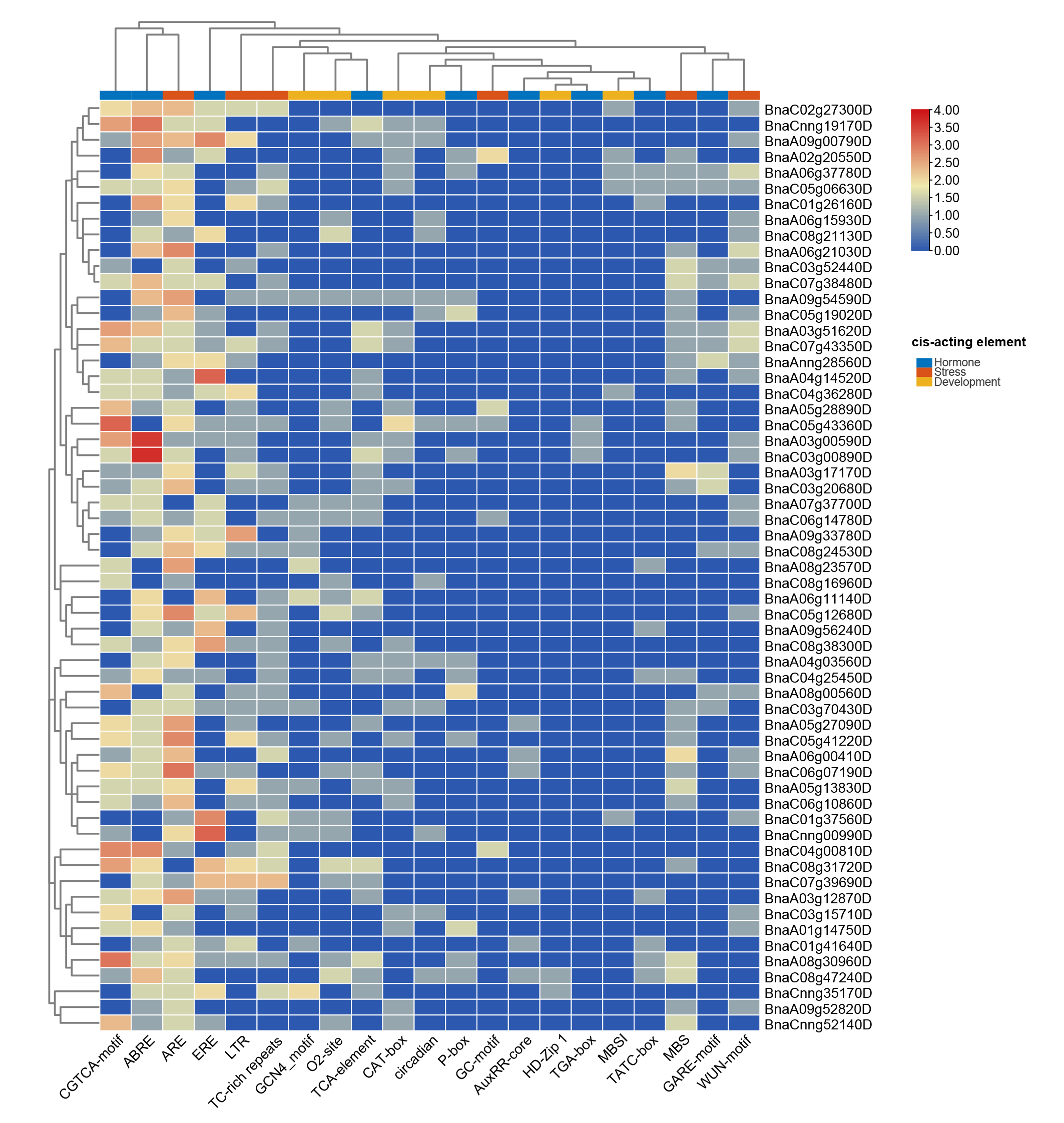

Supplement: Supplementary Figure 1 — Amount of cis-acting regulatory elements in promoters of SR genes in B. napus. Elements numbers were processed with log2 normalization. The color scale represented amounts from low (blue color) to high (red color). [file Image_1.JPEG]

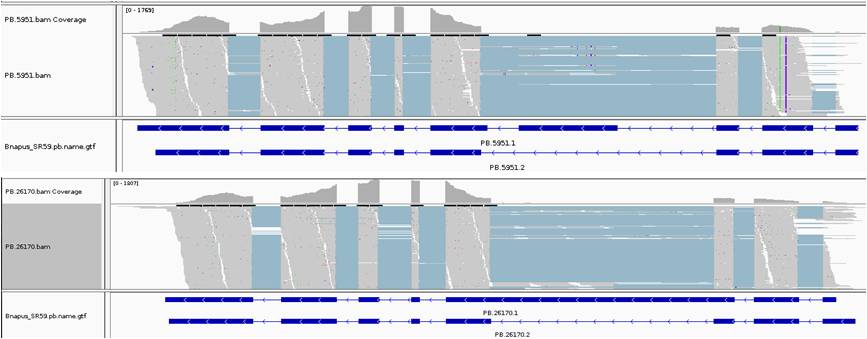

Supplement: Supplementary Figure 2 — The alignment information of BnaA04g03560D and BnaC04g25450D. [file Image_2.JPEG]

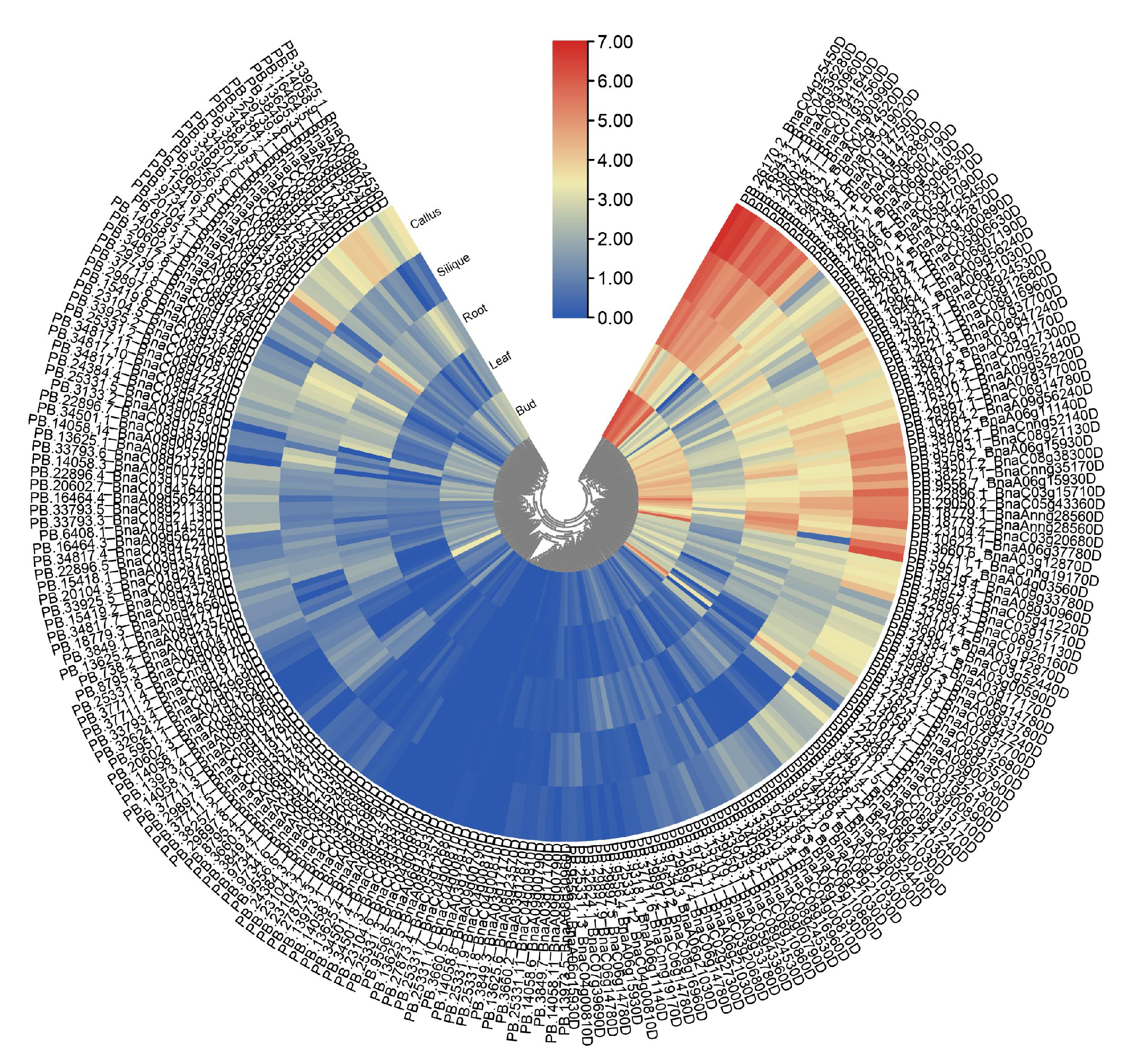

Supplement: Supplementary Figure 3 — Heatmap representation of transcripts of SR genes in different tissues. Expression data were processed with log2 normalization. The color scale represented relative expression levels from low (blue color) to high (red color). [file Image_3.JPEG]

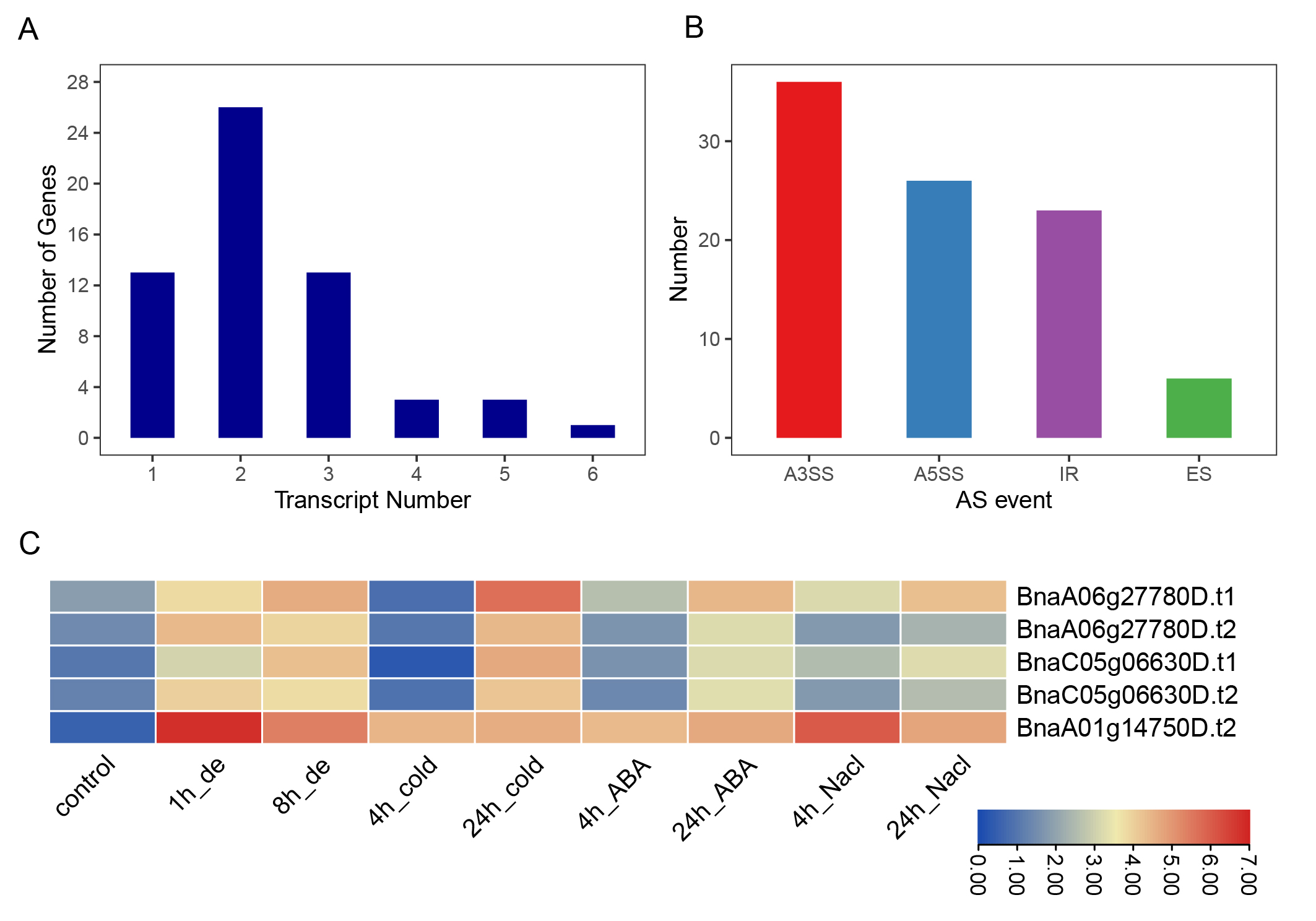

Supplement: Supplementary Figure 4 — Splicing profiles of SR genes in B. napus under abiotic stress condition. (A) Distribution of genes that produced one or more transcripts from RNA-Seq data. (B) Classification of AS events from RNA-Seq data. IR, intron retention; A3SS, alternative 3′ splice site; A5SS, alternative 5′ splice site; ES, exon skipping. (C) Five transcripts were obviously induced by all four stresses. Expression data were processed with log2 normalization. The color scale represented relative expression levels from low (blue color) to high (red color). [file Image_4.JPEG]

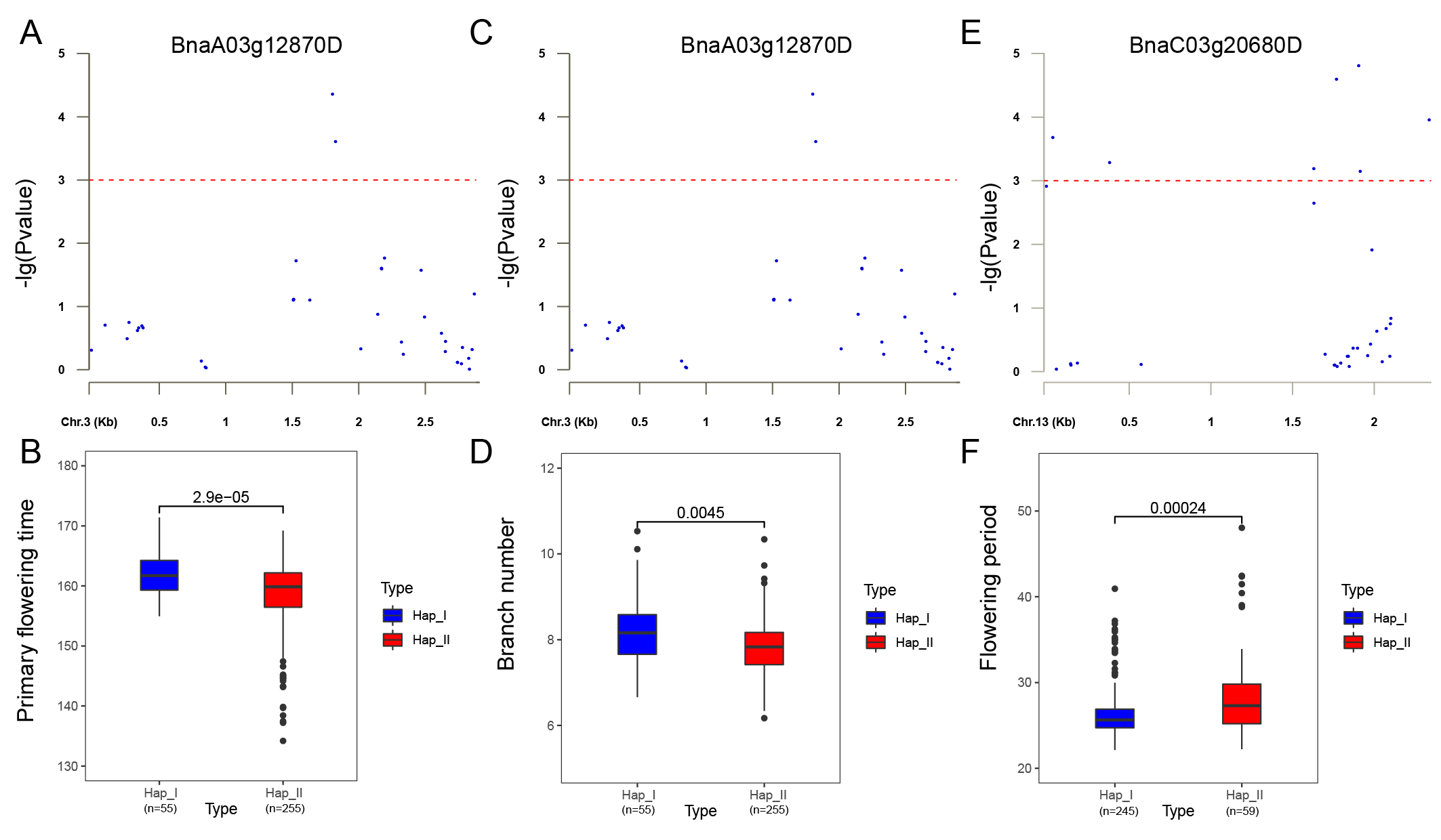

Supplement: Supplementary Figure 5 — Association mapping analysis of SR genes in 324 core collections of B. napus germplasm. (A,B) BnaA03g12870D was significantly associated with primary flowering time. (C,D) BnaA03g12870D was significantly associated with branch number. (E,F) BnaC03g20680D was significantly associated with the flowering period. [file Image_5.JPEG]
